# Supplementary material for: Fast imaging of millimeter-scale areas with beam deflection transmission electron microscopy
Source: Nat Commun. 2024 Aug 10;15:6860. doi: 10.1038/s41467-024-50846-4 (PMC11316758; doi:10.1038/s41467-024-50846-4)
Supplement: Supplementary file 1 — Supplementary Information [file 41467_2024_50846_MOESM1_ESM.pdf]

Supplementary figures and methods

Fast imaging of millimeter-scale areas with beam deflection transmission electron microscopy

Zhihao Zheng[1], Christopher S. Own[2], Adrian A. Wanner[1,3], Randal A. Koene[2], Eric W. Hammerschmith[1], William M. Silversmith[1], Nico Kemnitz[1], Ran Lu[1], David W. Tank[1], H. Sebastian Seung[1,*]

[1]Princeton Neuroscience Institute, Princeton University, Princeton, NJ, USA
[2]Voxa, Seattle, WA, USA

[3]Present address: Paul Scherrer Institute, Villigen, Switzerland
*Correspondence to sseung@princeton.edu

Supplementary Fig. 1

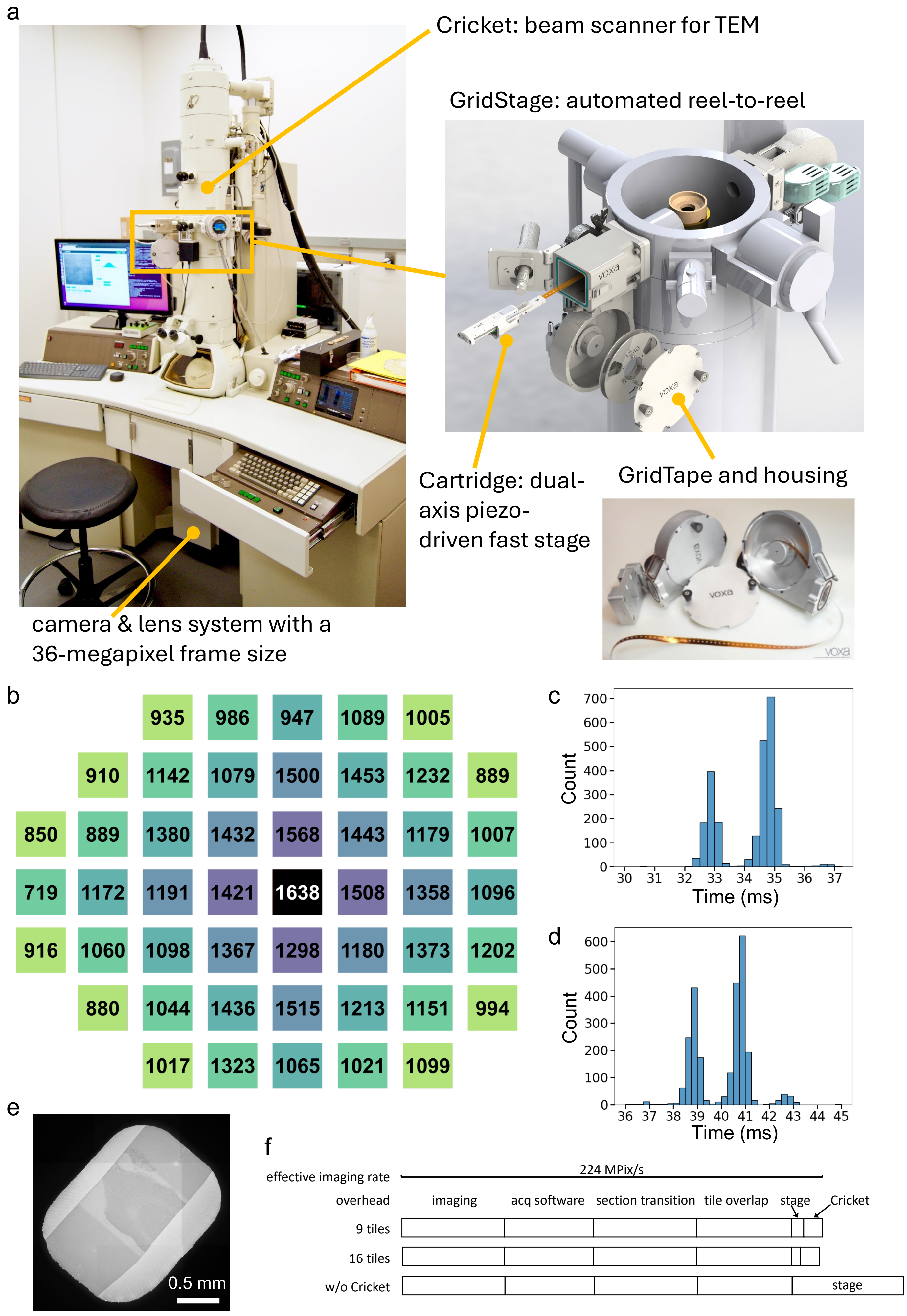

Supplementary Fig. 1. A bdTEM for high-throughput imaging. a, A refurbished JEOL 1200EX-II TEM with 120 KV accelerating voltage. The custom modifications include a beam deflection mechanism (Cricket, Voxa), an advanced reel-to-reel tape translation system with a dual-axis piezo-driven fast stage (GridStage, Voxa), and a high speed CMOS camera (CB500MG-CM, XIMEA) with improved lens design (NanoSprint50M-AV, AMT). **b,** Integrated spectral-SNR values across subtiles between the frequencies of 10^{-2} and 10^{-1} (1/nm) from Figure 2d. **c - d**, Distributions of stage motion time in x (**c**) and y (**d**) axes, with 2,500 counts of measurements for either axis. x-axis, mean 34.18 ms, s.d. 0.97 ms; y-axis, mean 40.09 ms, s.d. 1.14 ms. The stage is designed to carry less mass on the x-axis and therefore travels faster in the x direction. During imaging, an extra 10 ms of settling time is added for each movement to ensure stability (Methods). **e,** Overview of a section on a 2 mm x 1.5 mm slot imaged using Cricket at low magnification. **f,** Comparison of imaging overheads between 9 tiles, 16 tiles per supertile and without Cricket. Performances for imaging, acquisition software, section transition, and tile overlap are based on measurements in Figure 1e. Stage (or Cricket) overheads are calculated by multiplying the number of movements (or deflections) by their respective durations.

Supplementary Fig. 2

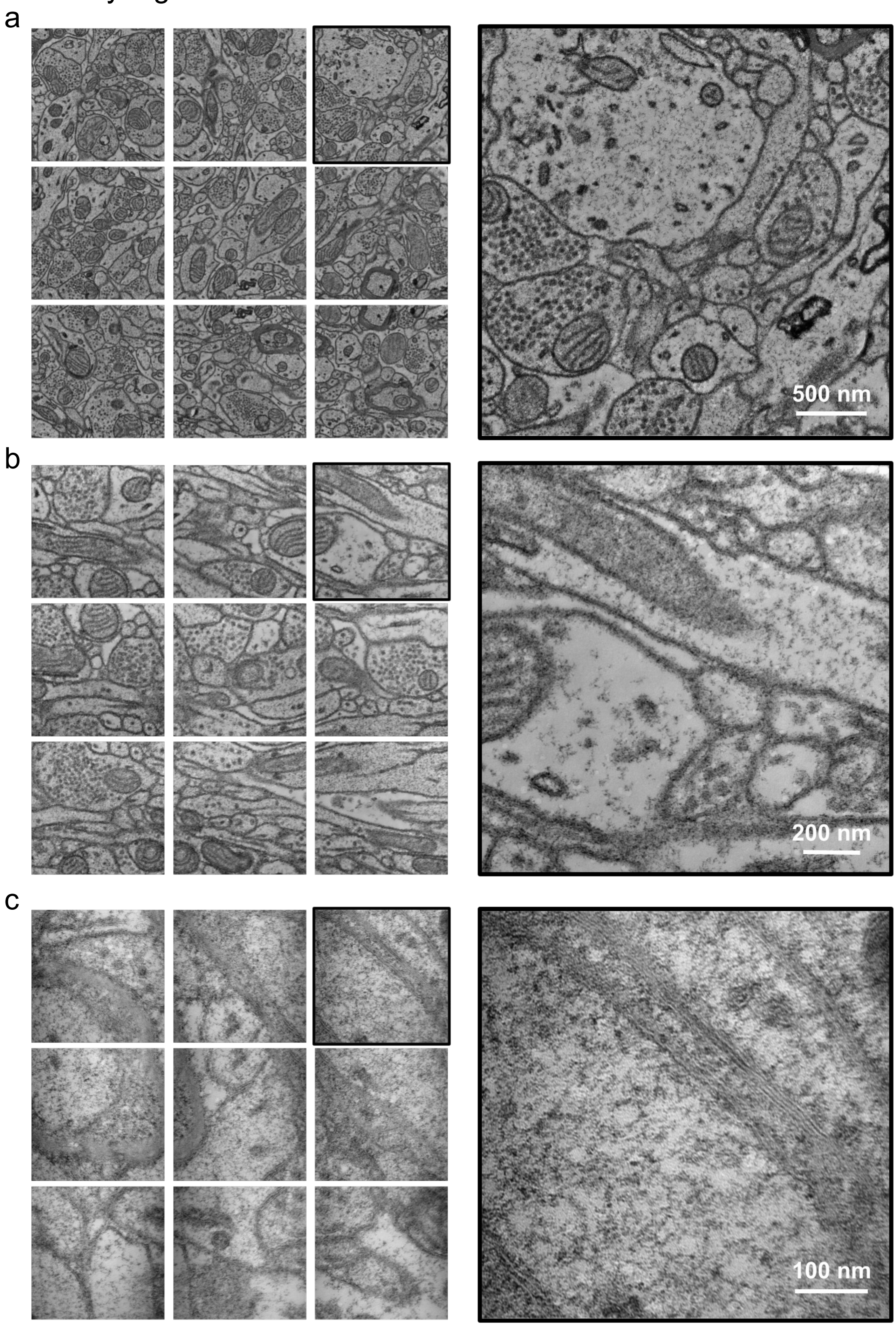

Supplementary Fig. 2. High-resolution imaging with bdTEM. a - c, High-resolution electron micrographs of a mouse cortex sample imaged with Cricket. Each of three supertiles are respectively imaged with magnifications of 20,000 (**a**, 0.48 nm/pix), 40,000 (**b**, 0.24 nm/pix), and 100,000 (**c**, 0.093 nm/pix) in the JEOL 1200EX-II TEM. A magnified view of the upper right subtiles is shown on the right-hand side.

Supplementary Fig. 3

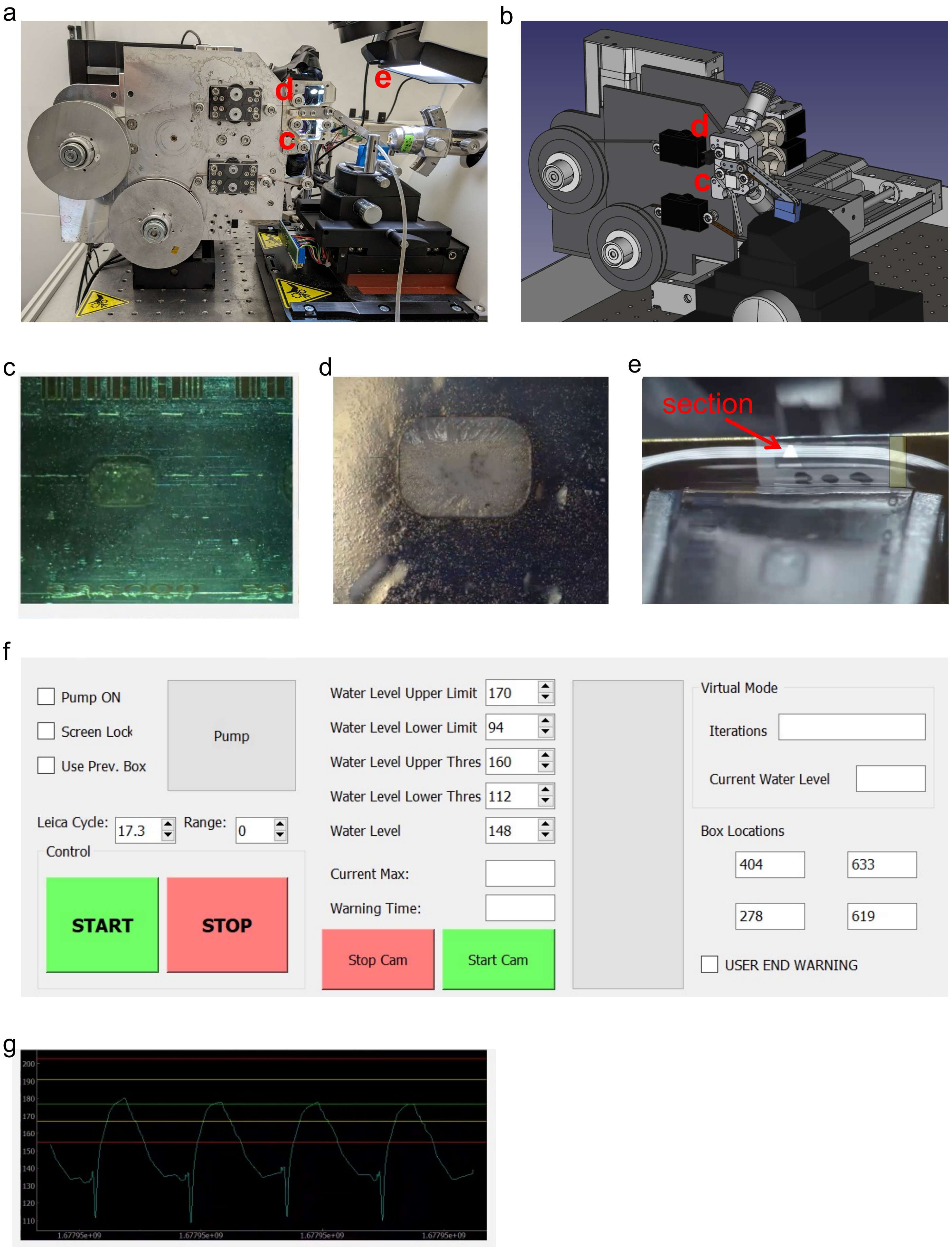

Supplementary Fig. 3 (cont.)

h

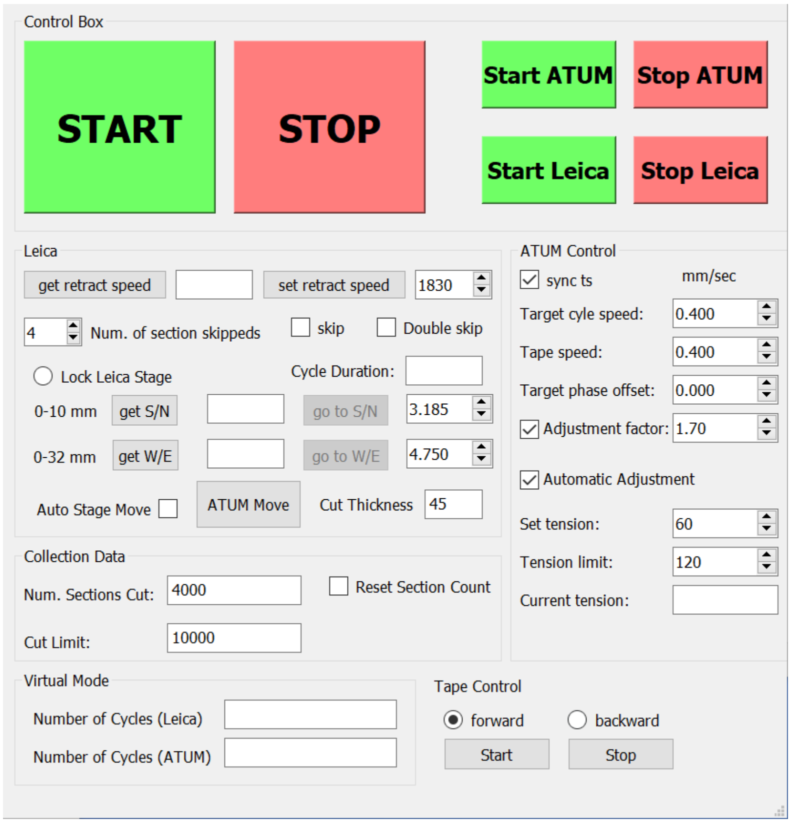

i

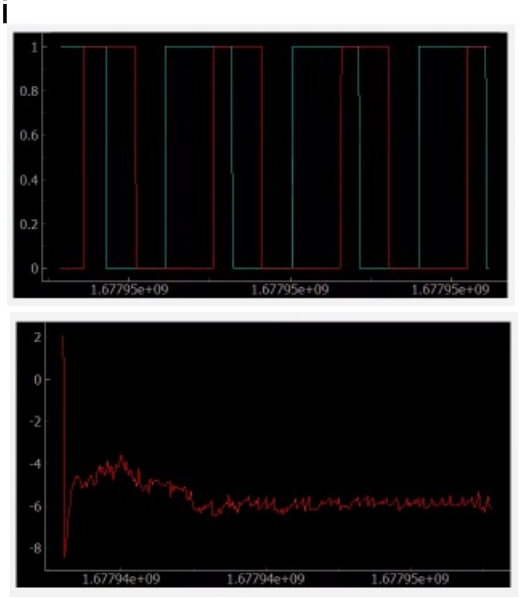

j

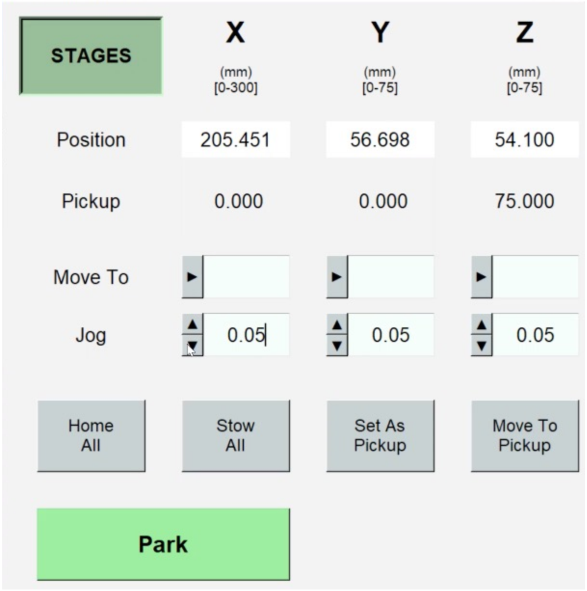

Supplementary Fig. 3. GridTape compatible automated ultramicrotome.
a - b, A photograph and a 3D model of an ATUMtome (RMC/Boeckeler) modified to be compatible with GridTape and Leica UC7 ultramicrotome. **c**, An image from the before-sectioning camera shows a GridTape aperture with a barcode on the top. **d**, An image from the after-sectioning camera shows drying and outline of a section on the film in the aperture. **e**, An image from the UC7 camera module shows the light reflection of an ultrathin section (45 nm) on the water, and pixel intensity inside the yellow rectangle is used to provide feedback to monitor water level continuously. **f - g**, Water control module and real-time plot of pixel intensity as an indication of water level over time. **h - i**, Tape control module with real-time plot of cycle time for each slot. **j**, Stage control module is used to control a three-axis stage that supports the base plate.

Supplementary Fig. 4

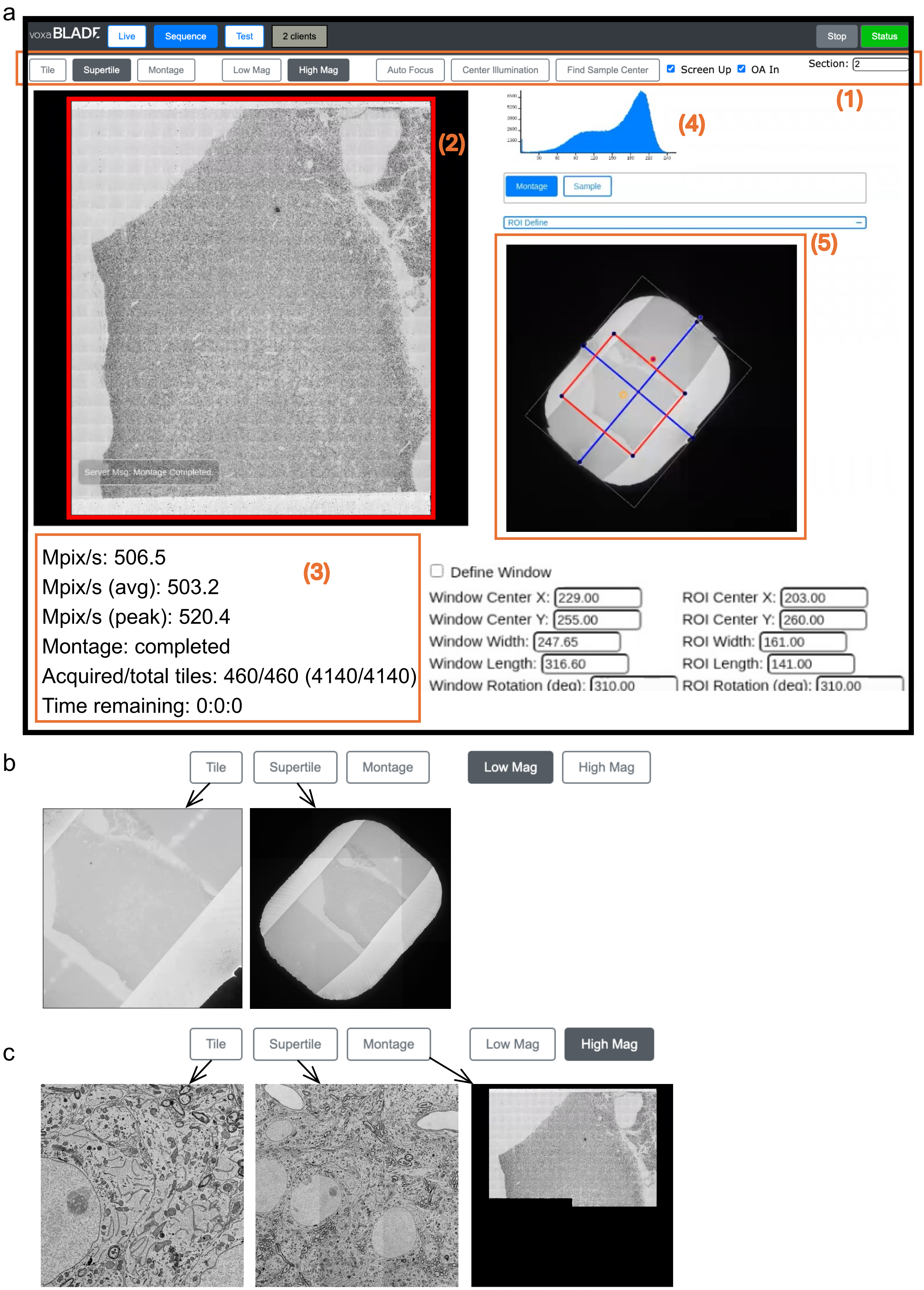

Supplementary Fig. 4. TEM imaging software. a, Control panels in the Blade imaging software. (1) Scope operation: Screen Up, controls the microscope viewing screen; OA in, controls the objective aperture; for other buttons, refer to Supplementary Fig. 4b-c, & 5. (2) Image display: see b - c. (3) Imaging status: real-time, average, and peak imaging speeds in megapixels per second (Mpix/s); Acquired/total, number of acquired tiles (or supertiles) out of total number of tiles (and supertiles) per section; t_rem, time remaining in imaging the current section. (4) Real-time image histogram. (5) ROI definition: blue cross ('Window') defines the location, size, and rotation of the slot (2 x 1.5 mm^2); red square ('ROI', region-of-interest) defines the location, size, and rotation of the ROI for high-resolution montage imaging. **b,** When Low Magnification (50x) is activated in the TEM, the Image display panel, (2) in **a**, depicts either a single undeflected tile or a supertile composed of 9 subtiles. **c,** When High Magnification (2900x) is activated, the image display panel depicts a single undeflected tile, a supertile composed of 9 subtiles, or the real-time progression of a high-resolution montage imaging session.

Supplementary Fig. 5

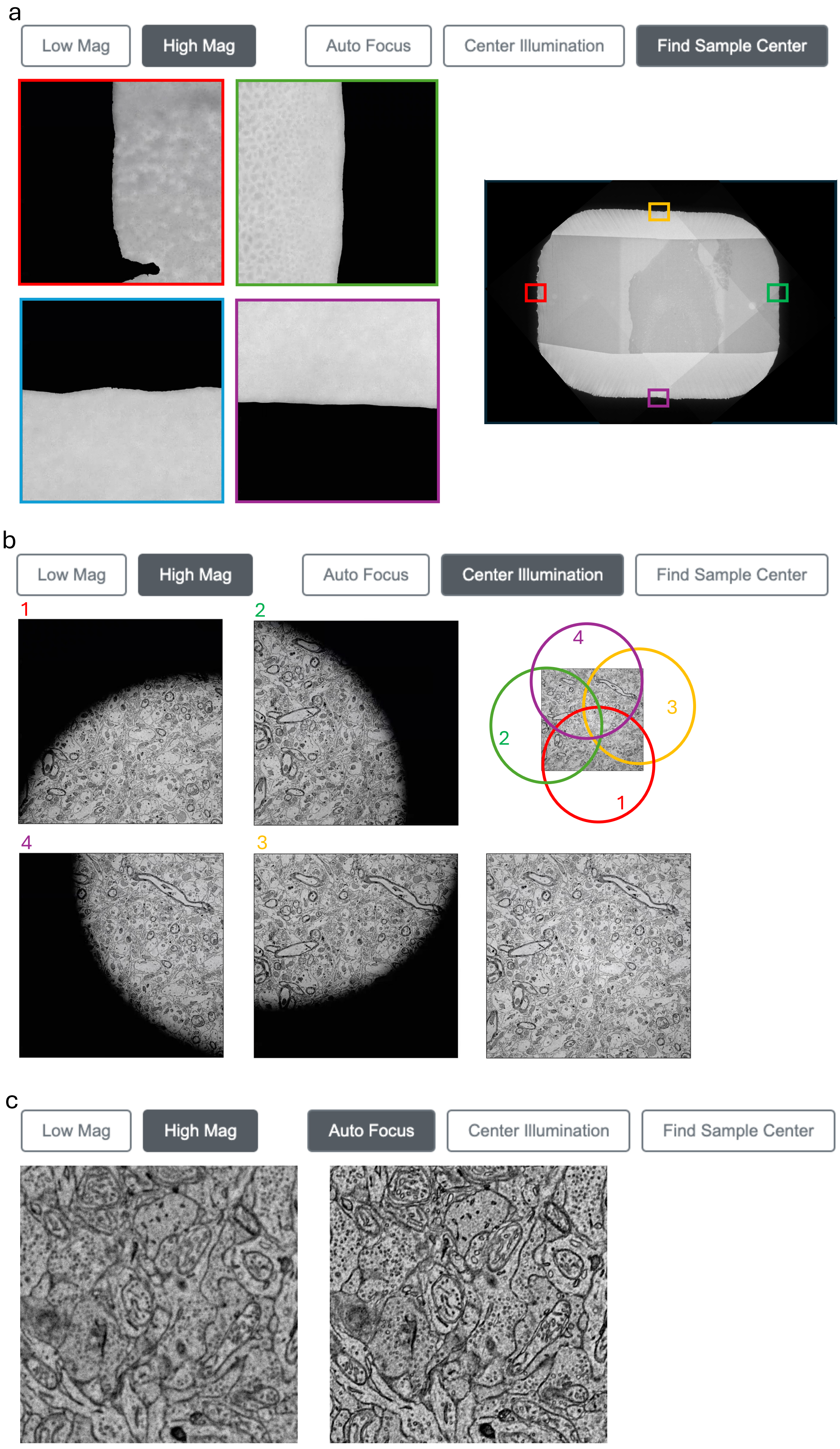

Supplementary Fig. 5. Automated functionality of TEM imaging software. a, Find Sample Center: this function identifies slot coordinates for defining ROI in subsequent montage imaging. The stage scans along four cardinal directions, detecting slot edges by analyzing the integrated histogram intensity of detected patches in the images. After pinpointing the positions of all four edges, the slot center is calculated from their midpoints. **b,** Center Illumination: this function calibrates the electron beam's illumination center, correcting for potential offsets due to lens hysteresis. By deflecting the beam along four directions, it identifies the point where half of the field of view is illuminated through the analysis of integrated histogram intensity from detected patches in the images. The center is determined by computing the midpoint of these deflections. **c**, Auto focus: this function is used to determine the optimal focus of the section for high-resolution imaging, based on a fast Fourier transform algorithm.

Supplementary Methods

Quantification of imaging rates. In general, imaging rates (Fig. 1d, Table 1) are computed as the number of pixels that are acquired in a given amount of time to image a section. Burst imaging rates are calculated from the number of pixels in a frame over camera exposure time per frame (6,000 x 6,000 pixels in 40 ms). Section imaging rates are calculated from the total number of pixels of all images (i.e. number of pixels in a tile multiplied by number of tiles) over time, from the first image to the last image of a section acquisition. Sources of overhead for section imaging rates include imaging, stage motion, beam deflection, computational overhead of the acquisition software, and computing basic image statistics (Fig. 1d). Net imaging rates are computed from the same number of pixels as in section imaging rate, including all overhead from section imaging, but additionally includes overhead for section transition (e.g. low-magnification overview of sections on GridTape, ROI extraction, autofocus, beam centering). Effective imaging rates are calculated from the total number of pixels of a montage acquisition with overlap pixels only counted once, over the same overhead as in the net imaging rate. Time for a section acquisition varies from section to section depending on variable computational overhead in the acquisition software. Peak imaging rate is taken from an acquisition with the shortest imaging time. Average transition time is the average taken from 10 consecutive automatic acquisitions.

The distribution of section imaging rates (Fig. 1e) is collected from acquisitions of 472 sections over the course of several days of imaging. Because the section imaging rate is calculated by the total number of pixels divided by time span for a given section, the variability of acquisition time span is directly reflected in the variability of section imaging rates. Each section encapasses 748 supertiles, with a size of approximately 1.9 x 1.2 mm^2. Based on these data, the mean and standard deviation of section imaging rates are, respectively, 397.1 MPix/s and 5.5 MPix/s. The section imaging rates are then multiplied by the total number of pixels (i.e. number of pixels per tile multiplied by the tile count) to calculate the time span for imaging a 1 mm^2 section. Based on these data, the mean and standard deviation of imaging time for a 1 mm^2 section is 6.58 mins and 0.9 mins, respectively. These values are empirically consistent with a typical imaging time for a 1 mm^2 area.

The calculation of stage motion overhead involves the multiplication of the average settling time for the x and y axes (34 ms and 40 ms, respectively, Supplementary Fig. 1c-d), by the total number of x and y movements required for imaging a section. Additionally, an extra 10 ms is added for each movement to cover edge cases and ensure sufficient settle time for stage. The 10 ms is used as an imaging parameter in our regular imaging procedures. A 1 mm^2 section consists of 484 supertiles, including 462 x-axis steps (34 ms per step) and 22 y-axis steps (40 ms per step), with an extra 10 ms per step. The total stage motion time is 21.5 seconds. The fraction of stage motion overhead is therefore 4% out of 8.8 mins (6.6 mins imaging time and 2.2 transition time). Given the standard deviation of x and y stage movements are 1 ms, which is already taken into account in the extra 10 ms (Table 1). The overhead for stage motion for a whole section is therefore quite stable.

Similarly, beam deflection overhead involves multiplication of per-tile settle time by the number of subtiles. We have empirically used a Cricket settle time of 8 ms per subtile. Given beam deflection operates on the order of several milliseconds or much less[1-3], instances of fuzzy images caused by beam deflection are exceedingly rare with 8 ms. The total Cricket settle time for a 1 mm^2 section is therefore 31 seconds (8 ms per subtile, for a total of 484 supertiles, each with 8 beam-deflected subtiles), which represent 6% out of total time per section (8.8 mins).

Overall, the time required for stage motion and beam deflection during the imaging of a section are determined by the total tile count for a section and tends to remain stable. The variances of their fractions are therefore determined by the variances of total section imaging time (Fig. 1e).

Imaging duty cycle is computed as the fraction of time for image acquisition (number of tiles multiplied by exposure time per tile) out of total time per section. For example, at 3 nm/Pixel the image acquisition time is 2.9 minutes (4,356 tiles at 40 ms per tile), which is 32.9% out of total time per section (8.8 minutes). Similarly, the imaging duty cycle at 4 nm/Pixel is 31%. In comparison, the peak performance of the previous state of the art system[4] has an imaging duty cycle of 15% (2,600 tiles at 50 ms per tile out of 14 minutes of total time).

1. Sadorf, H. & Kratz, H. A. Plug-in fast electron beam chopping system. Rev. Sci. Instrum. 56, 567–571 (1985).
2. Zhang, L., Garming, M. W. H., Hoogenboom, J. P. & Kruit, P. Beam displacement and blur caused by fast electron beam deflection. Ultramicroscopy 211, 112925 (2020).
3. Gesley, M. An electron optical theory of beam blanking. Rev. Sci. Instrum. 64, 3169–3190 (1993).
4. Yin, W. et al. A petascale automated imaging pipeline for mapping neuronal circuits with high-throughput transmission electron microscopy. Nat. Commun. 11, 4949 (2020).
