# Supplementary material for: Fast imaging of millimeter-scale areas with beam deflection transmission electron microscopy
Source: Nat Commun. 2024 Aug 10;15:6860. doi: 10.1038/s41467-024-50846-4 (PMC11316758; doi:10.1038/s41467-024-50846-4)
Supplement: Supplementary file 3 — Description of Additional Supplementary Files [file 41467_2024_50846_MOESM3_ESM.pdf]

Description of Additional Supplementary Files

Supplementary Movie Legends:

Supplementary Movie 1. Aligned images across 55 sections acquired at 120 ms exposure time.

Supplementary Movie 2. Aligned images across 20 sections acquired at 40 ms exposure time.

Supplementary Movie 3. Screen recording of an acquisition at a peak imaging rate.
